# Medication for Opioid Use Disorder During Pregnancy — Maternal and Infant Network to Understand Outcomes Associated with Use of Medication for Opioid Use Disorder During Pregnancy (MAT-LINK), 2014–2021

**DOI:** 10.15585/mmwr.ss7203a1

**Published:** 2023-05-05

**Authors:** Kathryn Miele, Shin Y. Kim, Rachelle Jones, Juneka H. Rembert, Elisha M. Wachman, Hira Shrestha, Michelle L. Henninger, Teresa M. Kimes, Patrick D. Schneider, Vaseekaran Sivaloganathan, Katherine A. Sward, Vikrant G. Deshmukh, Pilar M. Sanjuan, Jessie R. Maxwell, Neil S. Seligman, Sarah Caveglia, Judette M. Louis, Tanner Wright, Carolyne Cody Bennett, Caitlin Green, Nisha George, Lucas Gosdin, Emmy L. Tran, Dana Meaney-Delman, Suzanne M. Gilboa

**Affiliations:** ^1^Division of Birth Defects and Infant Disorders, National Center on Birth Defects and Developmental Disabilities, CDC, Atlanta, Georgia; ^2^Public Health Informatics Institute, Decatur, Georgia; ^3^Boston Medical Center, Boston, Massachusetts; ^4^Center for Health Research, Kaiser Permanente Northwest, Portland, Oregon; ^5^The Ohio State University, Columbus, Ohio; ^6^University of Utah, Salt Lake City, Utah; ^7^University of New Mexico Health Sciences Center, Albuquerque, New Mexico; ^8^University of Rochester, Rochester, New York; ^9^University of South Florida, Tampa, Florida; ^10^Eagle Global Scientific, Atlanta, Georgia; ^11^Epidemic Intelligence Service, CDC, Atlanta, Georgia

## Abstract

**Problem:**

Medication for opioid use disorder (MOUD) is recommended for persons with opioid use disorder (OUD) during pregnancy. However, knowledge gaps exist about best practices for management of OUD during pregnancy and these data are needed to guide clinical care.

**Period Covered:**

2014–2021.

**Description of the System:**

Established in 2019, the Maternal and Infant Network to Understand Outcomes Associated with Medication for Opioid Use Disorder During Pregnancy (MAT-LINK) is a surveillance network of seven clinical sites in the United States. Boston Medical Center, Kaiser Permanente Northwest, The Ohio State University, and the University of Utah were the initial clinical sites in 2019. In 2021, three clinical sites were added to the network (the University of New Mexico, the University of Rochester, and the University of South Florida). Persons receiving care at the seven clinical sites are diverse in terms of geography, urbanicity, race and ethnicity, insurance coverage, and type of MOUD received. The goal of MAT-LINK is to capture demographic and clinical information about persons with OUD during pregnancy to better understand the effect of MOUD on outcomes and, ultimately, provide information for clinical care and public health interventions for this population. MAT-LINK maintains strict confidentiality through robust information technology architecture. MAT-LINK surveillance methods, population characteristics, and evaluation findings are described in this inaugural surveillance report. This report is the first to describe the system, presenting detailed information on funding, structure, data elements, and methods as well as findings from a surveillance evaluation. The findings presented in this report are limited to selected demographic characteristics of pregnant persons overall and by MOUD treatment status. Clinical and outcome data are not included because data collection and cleaning have not been completed; initial analyses of clinical and outcome data will begin in 2023.

**Results:**

The MAT-LINK surveillance network gathered data on 5,541 reported pregnancies with a known pregnancy outcome during 2014–2021 among persons with OUD from seven clinical sites. The mean maternal age was 29.7 (SD = ±5.1) years. By race and ethnicity, 86.3% of pregnant persons were identified as White, 25.4% as Hispanic or Latino, and 5.8% as Black or African American. Among pregnant persons, 81.6% had public insurance, and 84.4% lived in urban areas. Compared with persons not receiving MOUD during pregnancy, those receiving MOUD during pregnancy were more likely to be older and White and to have public insurance. The evaluation of the surveillance system found that the initial four clinical sites were not representative of demographics of the South or Southwest regions of the United States and had low representation from certain racial and ethnic groups compared with the overall U.S. population; however, the addition of three clinical sites in 2021 made the surveillance network more representative. Automated extraction and processing improved the speed of data collection and analysis. The ability to add new clinical sites and variables demonstrated the flexibility of MAT-LINK.

**Interpretation:**

MAT-LINK is the first surveillance system to collect comprehensive, longitudinal data on pregnant person–infant dyads with perinatal outcomes associated with MOUD during pregnancy from multiple clinical sites. Analyses of clinical site data demonstrated different sociodemographic characteristics between the MOUD and non-MOUD treatment groups.

**Public Health Actions:**

MAT-LINK is a timely and flexible surveillance system with data on approximately 5,500 pregnancies. Ongoing data collection and analyses of these data will provide information to support clinical and public health guidance to improve health outcomes among pregnant persons with OUD and their children.

## Introduction

From 1999 to 2014, the prevalence of opioid use disorder (OUD) among pregnant women in the United States quadrupled from 1.5 to 6.5 per 1,000 delivery hospitalizations (*1*). Although medication for OUD (MOUD), such as methadone and buprenorphine, is recommended for persons with OUD during pregnancy, knowledge gaps exist about risks for and benefits of each MOUD regimen and whether certain medications or prescribing patterns result in improved outcomes (*2*,*3*). Other treatment options (e.g., naltrexone and medically supervised opioid withdrawal) are not first-line treatments and are less frequently used during pregnancy (*2*). Persons also might use nonpharmacologic treatments, switch between different types of MOUD, and have active substance use while taking MOUD during pregnancy (*4*). Although polysubstance use (i.e., the use of multiple substances) during pregnancy is common, existing data sets have limited ability to properly define or measure use of multiple substances (*5*,*6*). Because of these complexities, large multisite collaboratives with comprehensive data are needed to conduct robust analyses of outcomes and assess the effect of mediating and moderating factors, including polysubstance exposure, social determinants of health, timing of MOUD during pregnancy, and availability of other services for OUD management.

This report provides a detailed description of a sentinel surveillance system for MOUD during pregnancy, including data sources, types of variables, methods for secure data transfer and storage, and the process for making data available for future analyses. In addition, selected population characteristics by MOUD status from clinical sites are described and the results of a surveillance evaluation are presented. Linked data on pregnant person–infant dyads might be used by public health professionals, clinicians, and policymakers seeking to improve the care of pregnant persons with OUD.

## Methods

**FIGURE 1:**
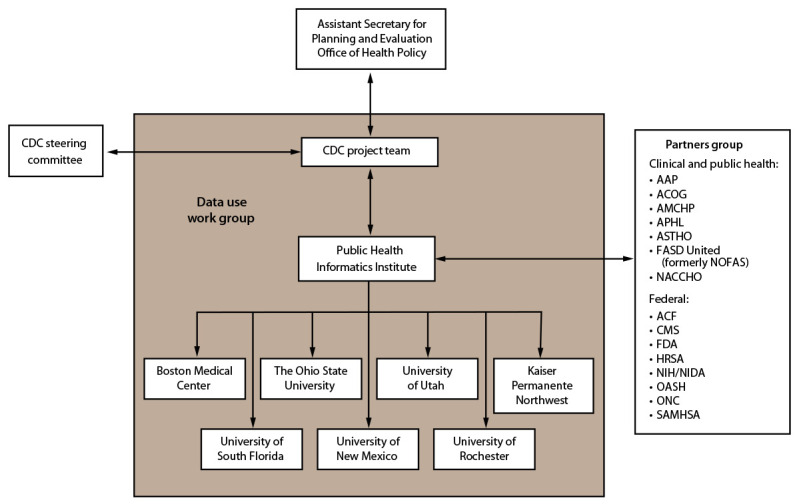
Organizational structure and governance — Maternal and Infant Network to Understand Outcomes Associated with Medication for Opioid Use Disorder During Pregnancy (MAT-LINK), 2019–2023 **Abbreviations:** AAP = American Academy of Pediatrics; ACF = Administration for Children and Families; ACOG = American College of Obstetricians and Gynecologists; AMCHP = Association of Maternal and Child Health Programs; APHL = Association of Public Health Laboratories; ASTHO = Association of State and Territorial Health Officials; CMS = Centers for Medicare & Medicaid Services; FASD United = Fetal Alcohol Spectrum Disorders United (formerly NOFAS); FDA = Food and Drug Administration; HRSA = Health Resources and Services Administration; NACCHO = National Association of County and City Health Officials; NIH/NIDA = National Institutes of Health/National Institute on Drug Abuse; OASH = Office of the Assistant Secretary for Health; ONC = Office of the National Coordinator for Health Information Technology; SAMHSA = Substance Abuse and Mental Health Services Administration.

CDC, in collaboration with the Public Health Informatics Institute (PHII) and with funding from the Assistant Secretary for Planning and Evaluation (ASPE)’s Office of the Secretary Patient-Centered Outcomes Research Trust Fund (OS-PCORTF), established the Maternal and Infant Network to Understand Outcomes Associated with Medication for Opioid Use Disorder During Pregnancy (MAT-LINK) in 2019 (Figure 1). MAT-LINK is a surveillance network of clinical sites that collect data on persons with OUD during pregnancy. In this report, the term “maternal” is used to identify the person who is pregnant or postpartum. Pregnancy is not equated with the decision to parent, nor do all parents who give birth identify as mothers.

CDC is responsible for day-to-day project management and surveillance network design. CDC also leads data collection and cleaning, develops analytic protocols and guidance documents, and monitors project performance and spending. PHII, the implementation partner, subcontracts with the clinical sites and manages the surveillance infrastructure according to relevant federal regulations under CDC’s direction. PHII also ensures the integrity and safeguarding of data and provides technical assistance to the clinical sites.

The MAT-LINK partners group comprises federal, clinical, and public health partners. Federal partners provide subject matter expertise and individual input into the implementation of MAT-LINK. The clinical and public health partners consult with the CDC project team to share perspectives from their relevant constituencies. A CDC steering committee composed of leadership and subject matter experts from multiple CDC centers provides guidance and oversight of MAT-LINK.

### Participating Clinical Sites

MAT-LINK was established in 2019 and initially comprised four clinical sites: Boston Medical Center, Kaiser Permanente Northwest, The Ohio State University, and the University of Utah. These clinical sites were selected because they had an advanced and robust data infrastructure, clinical care protocols that included multiple MOUD regimens, and the capacity to capture postpartum and childhood outcome data up to age 2 years. They also were required to demonstrate their ability to integrate or link maternal and child data (i.e., dyads) as well as their authority to access and share these data with CDC. In 2021, with more funding for expansion from ASPE’s OS-PCORTF, three additional clinical sites were selected: the University of New Mexico, the University of Rochester, and the University of South Florida. This expansion of MAT-LINK also included the collection of childhood data through age 6 years to enable assessment of neurodevelopmental outcomes and outcomes for school-age children.

### Case Ascertainment

The inclusion criteria for MAT-LINK were all known pregnancy outcomes from January 1, 2014, through August 31, 2021, and an *International Classification of Diseases*, *Ninth* or *Tenth Revision, Clinical Modification* (ICD-CM) code for OUD diagnosis during that pregnancy (Table 1). Clinical site staff members worked with their principal investigators and clinical care teams to identify all eligible pregnancies. Each clinical site then assigned a unique identifier to each dyad of linked maternal and child electronic health record (EHR) data before submitting data. If a duplicate identifier was generated, clinical site staff members informed CDC immediately, deleted any previously submitted duplicative data from the data repositories, and excluded the duplicated dyad identifier from any future data submissions. CDC does not receive personally identifiable data (e.g., names or addresses) but does receive potentially identifiable and sensitive data (e.g., birth dates and hospital admission dates). However, the unique dyad identifiers do not include personal, institutional, or geographic information.

**TABLE 1:** *International Classification of Diseases, Tenth Revision, Clinical Modification* (ICD-10-CM) and *International Classification of Diseases, Ninth Revision, Clinical Modification* (ICD-9-CM) diagnoses relating to opioid use disorder during the peripartum

Code and category	Description
**ICD-10-CM**	
F11	Opioid-related disorders
F11.1X*	Opioid abuse
F11.2X*	Opioid dependence
F11.9X*	Opioid use, unspecified
F11.10	Opioid abuse, uncomplicated
F11.11	Opioid abuse, in remission (only for dyads receiving MOUD during pregnancy)
F11.12X*	Opioid abuse with intoxication
F11.13	Opioid abuse with withdrawal
F11.14	Opioid abuse with opioid-induced mood disorder
F11.15X*	Opioid abuse with opioid-induced psychotic disorder
F11.18X*	Opioid abuse with other opioid-induced disorder
F11.19	Opioid abuse with unspecified opioid-induced disorder
F11.21	Opioid dependence, in remission (only used for dyads receiving MOUD during pregnancy)
F11.22X*	Opioid dependence with intoxication
F11.23	Opioid dependence with withdrawal
F11.24	Opioid dependence with opioid-induced mood disorder
F11.25X*	Opioid dependence with opioid-induced psychotic disorder
F11.28X*	Opioid dependence with other opioid-induced disorder
F11.29	Opioid dependence with unspecified opioid-induced disorder
F11.92X*	Opioid use, unspecified with intoxication
F11.93	Opioid use, unspecified with withdrawal
F11.94	Opioid use, unspecified with opioid-induced mood disorder
F11.95X*	Opioid use, unspecified with opioid-induced psychotic disorder
F11.98X*	Opioid use, unspecified with other specified opioid-induced disorder
F11.99	Opioid use, unspecified with unspecified opioid-induced disorder
O99.320	Drug use complicating pregnancy, unspecified trimester
O99.321	Drug use complicating pregnancy, first trimester
O99.322	Drug use complicating pregnancy, second trimester
O99.323	Drug use complicating pregnancy, third trimester
O99.324	Drug use complicating childbirth
O99.325	Drug use complicating the puerperium
T40.0X*	Poisoning by, adverse effect of, and underdosing of opium
T40.1X*	Poisoning by and adverse effect of heroin
T40.2X*	Poisoning by, adverse effect of, and underdosing of other opioids
T40.3X*	Poisoning by, adverse effect of, and underdosing of methadone
T40.4XX*	Poisoning by, adverse effect of, and underdosing of other synthetic narcotics
T40.6XX*	Poisoning by, adverse effect of, and underdosing of other and unspecified narcotics
**ICD-9-CM**	
648.30	Drug dependence of mother, unspecified as to episode of care or not applicable
304.00	Opioid-type dependence, unspecified
304.01	Opioid-type dependence, continuous
304.02	Opioid-type dependence, episodic
304.03	Opioid-type dependence, in remission (only for dyads receiving MOUD during pregnancy)
304.70	Combinations of opioid-type drug with any other drug dependence, unspecified
304.71	Combinations of opioid-type drug with any other drug dependence, continuous
304.72	Combinations of opioid-type drug with any other drug dependence, episodic
304.73	Combinations of opioid-type drug with any other drug dependence, in remission (only for dyads receiving MOUD during pregnancy)
305.50	Opioid abuse, unspecified
305.51	Opioid abuse, continuous
305.52	Opioid abuse, episodic
305.53	Opioid abuse, in remission (only for dyads receiving MOUD during pregnancy)
965.0X*	Poisoning by analgesics, antipyretics, and antirheumatics
E850.0	Accidental poisoning by heroin
E850.1	Accidental poisoning by methadone
E850.2	Accidental poisoning by other opiates and related narcotics
E935.0	Heroin causing adverse effects in therapeutic use
E935.1	Methadone causing adverse effects in therapeutic use
E935.2	Other opiates and related narcotics causing adverse effects in therapeutic use

**Abbreviation:** MOUD = medication for opioid use disorder.

* X is a placeholder character to indicate inclusion of all valid subcategories and codes.

### Data Sources, Collection, and Processing

**FIGURE 2:**
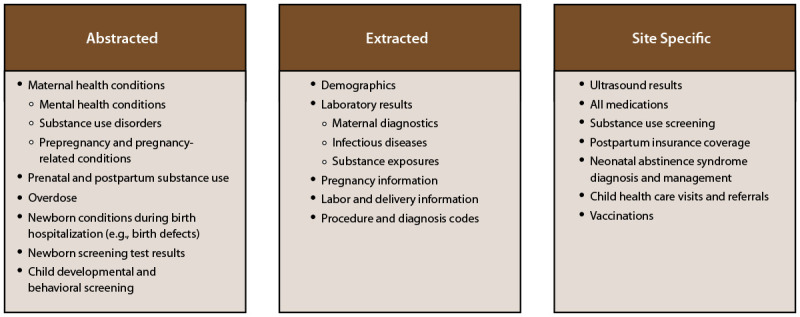
Variables by data collection method*^,†^ — Maternal and Infant Network to Understand Outcomes Associated with Medication for Opioid Use Disorder During Pregnancy (MAT-LINK), 2014–2021 * Certain variables designated as extracted (i.e., demographics, pregnancy information, and labor and delivery information) contain a mix of extracted and site-specific variables. ^†^ Variables designated as site specific are abstracted (i.e., manually retrieved from various chart sources) by certain sites and extracted (i.e., queried from relational data tables within an electronic health record) by others.

Data for MAT-LINK are collected by clinical sites from existing health services and medical records including EHRs, pharmaceutical management systems, laboratory records, public health reports, and state surveillance data. External sources for additional data might include MOUD-related visits from an outside clinic or administrative data sources. Health data at clinical sites are collected in accordance with state and local policies and procedures. Six clinical sites use Epic software and one clinical site uses Cerner software as their EHR system. Clinical sites have variations in how data are abstracted (i.e., manually retrieved from various chart sources), extracted (i.e., queried from relational data tables within an EHR), and reported (e.g., the use of custom data warehouses). Each clinical site has a unique approach to using and transforming its EHR data, including which fields are populated and how data are organized. Site-specific variations include which data are stored in structured format and which data are in text form (e.g., clinical notes or reports). Data were obtained by abstraction and extraction (Figure 2). Clinical sites used the data dictionary CDC provided to determine which variables would need to be abstracted or extracted at their individual clinical sites. A preset algorithm for data extraction was not used because of differences between sites in how data are entered and stored. In addition, a comparison group of pregnant persons not taking MOUD was sampled.

#### Information Technology Infrastructure

The information technology (IT) infrastructure for MAT-LINK was synchronously developed by CDC and PHII. The initial four clinical sites piloted the infrastructure using a collaborative approach for abstracted and extracted data. The main components included Microsoft SharePoint for project collaboration and support; Research Electronic Data Capture (REDCap) for entry of abstracted data; PHII-developed XML schemas for various data concepts; a MAT-LINK broker application to perform the extraction, transfer, and loading of abstracted and extracted data into a secure SQL server database; and a data extractor for generating and validating the schema-compliant XML files (Supplementary Figure, https://stacks.cdc.gov/view/cdc/126230) (*7*,*8*).

SharePoint is a web-based platform for sharing files and documents that allows the clinical sites to collaborate on data collection and receive support from the project team. CDC developed custom forms and workflows in SharePoint so users could submit questions confidentially with automated business processes to facilitate communication and collaboration.

Data entry and extraction are standardized by using REDCap instruments and the data extractor, respectively. A MAT-LINK–specific REDCap project, which contains approximately 1,000 variables, was developed for entry of data abstracted through medical chart review. A duplicate copy of the REDCap project is implemented at all MAT-LINK clinical sites. The data extractor is a customized tool with 28 XML schemas that support a standardized format for extracted data from data warehouses, EHR systems, and other sources. It includes data sets (e.g., ICD-CM codes and laboratory data) that can be automated for extraction from health care data systems. To reduce the need for previous XML knowledge at a clinical site, the data extractor package includes multiple support documents, such as a data dictionary, standard CSV templates for compiling the data, and validation scripts to generate XML files.

#### Data Quality and Sharing

After data abstraction and extraction, the clinical sites conduct both automated and manual checks to review the data for quality. These steps include internal quality control checks that perform dual entry verification for 10% of abstracted dyads and compare the data with what is expected from clinical experiences. The clinical sites also run their extracted data through a schema-specific validator in the data extractor to check for errors before submission. Any errors in the extracted EHR data are spot checked and rectified using chart reviews. Finalized data are shared with CDC via Secure Data Exchange, a secure file transfer platform. Data received are automatically subjected to a series of validation processes by the MAT-LINK broker before being deposited into a raw data server as a relational database. Next, CDC conducts a series of automated data quality checks. If no issues are detected, the data are imported into the production database. If the data set does not pass the data quality checks, the data team contacts the clinical site for clarification or correction of the data. Finally, CDC analysts conduct a manual review of the data to look for anomalies.

The IT architecture was designed to expand as the project needs develop. Additional topic areas and dyads can be added to the MAT-LINK REDCap project and data extractor. XML schemas in the data extractor that can be used by non-MOUD projects include demographics, procedures and diagnoses, medication schemas, and child follow-up schemas.

After data are collected and organized, each clinical site receives a final version of its own data (i.e., without data from other clinical sites). MAT-LINK partners and CDC are collaborating to analyze and report findings from the pooled data combined across clinical sites. Site-specific data will not be reported unless permission is provided by the clinical site.

A partial data set that is consistent with all institutional policies and the Assurance of Confidentiality will be available for analysis by external investigators after submission of a proposal to and approval from the National Center for Health Statistics’ Research Data Center. Guidance for external researchers interested in requesting access to a MAT-LINK data set will be available at https://www.cdc.gov/ncbddd/aboutus/mat-link.html.

#### Data Protection

Data provided to CDC within the MAT-LINK network are protected under an Assurance of Confidentiality (*9*). This activity was reviewed by CDC and was conducted consistent with applicable federal law and CDC policy.* Each clinical site received approval or exemption from the respective institutional review board before beginning data collection.

### Exposure Definitions and Comparison Group

For this analysis, MOUD included buprenorphine with or without naloxone, methadone, and naltrexone. Buprenorphine-based medications specifically approved for management of chronic pain were not included as MOUD; however, they were included with other medications as a potential co-exposure. All persons with a diagnosis of OUD during pregnancy who received MOUD in the pregnancy during the surveillance period were included in the MOUD group for analysis, including persons in remission or with a history of OUD before the pregnancy. Each person could have more than one pregnancy included in MAT-LINK and these pregnancies could be included in the same or different MOUD groups. Remission is designated by ICD-CM codes and includes persons with a previous diagnosis of OUD currently taking MOUD.

To provide a comparison group representative of pregnant persons with OUD who did not receive MOUD, clinical sites were asked to include extracted data on all pregnant persons with an active diagnosis of OUD during pregnancy who did not receive MOUD (the non-MOUD group). Clinical sites with >60 pregnancies among persons not taking MOUD were asked to provide a simple random sample of complete data (abstracted and extracted) on 60 dyads over the surveillance period.

### Description of Variables

MAT-LINK includes longitudinal data for the person who is pregnant from entry into prenatal care through 1 year postpartum and procedure and diagnosis codes (e.g., ICD-CM and Current Procedural Terminology [CPT] codes) up to 6 years postpartum. Longitudinal data for the child are collected from EHRs from birth through age 6 years. Pregnant person and child data are linked by the clinical sites. Determining the variables to collect was an iterative process that included literature reviews and input from subject matter experts. The ability to answer key analytic questions, ease of availability from existing data sources, and reasonable standardization across clinical sites were considered. The initial clinical sites provided a minimum of 25 records for all variables as pilot data; these were examined for data structure, quality, and compliance with data requirements. The additional clinical sites provided pilot data on key variables that were identified as difficult to ascertain from piloting efforts at the initial clinical sites. After pilot data were reviewed, all clinical sites provided final data.

#### Maternal History

Maternal history includes demographic and pregnancy-related data (e.g., encounter information, medications, laboratory data, ultrasounds, substance exposure laboratory results, infectious disease testing, procedures, and diagnoses). Race and ethnicity were categorized and reported based on standards specified by CDC’s Office of Management and Budget. Race categories are American Indian or Alaska Native (AI/AN), Asian, Native Hawaiian or other Pacific Islander (NH/OPI), Black or African American (Black), White, and other races; ethnicity categories are Hispanic or Latino (Hispanic) and not Hispanic or Latino (non-Hispanic). Asian and NH/OPI racial groups were consolidated to meet patient confidentiality requirements. Race and ethnicity are not mutually exclusive, and persons are accounted for in both categories. All multiracial and other race persons have been grouped under “other” races. Covariates, including psychosocial support, behavioral therapy, and peer support program participation as documented in the EHR, also are collected.

#### MOUD

Data collected on MOUD can be compared by timepoints during pregnancy. Information about how each clinical site provides MOUD to its patients will be used to understand possible differences between MOUD treatment groups. All clinical sites have data about MOUD integrated into their data collection system, regardless of whether the clinical site is the prescriber of the MOUD.

MOUD initiation, duration, and dosing patterns are included as well as other medications, including known teratogenic medications and medications associated with drug–drug interactions with MOUD. Data about inpatient or residential stays, substance use, and overdose events also are collected.

#### Delivery Birth Hospitalization

Variables related to pregnancy and delivery outcomes include pain management during and after labor; delivery type; newborn measurements; newborn care; laboratory data about infections and substance exposure; neonatal abstinence syndrome, neonatal opioid withdrawal syndrome, or both; and timing and other data on discharge, readmissions, and emergency department visits. Variables for pregnancy outcomes include live birth, intrauterine fetal death (≥20 weeks’ gestation), spontaneous abortion (<20 weeks’ gestation), termination, ectopic pregnancy, and other unspecified non-live births.

#### Child Follow-Up

Data are collected to examine short- and long-term outcomes for children, including physical growth and development, diagnoses of acute or chronic conditions, health care use, vaccinations, neurodevelopmental outcomes, medications, and referrals. Sources of this information include pediatric routine follow-up visits, acute care visits, and hospitalizations.

#### Postpartum

Additional data are collected postpartum. These variables include information about the person’s anxiety, depression, contraception, substance use screening, substance exposure laboratory results, MOUD, and inpatient or residential stays.

### Analysis

Selected maternal sociodemographic characteristics among pregnancies from the seven clinical sites by MOUD treatment status are presented. Variables include maternal age, race, ethnicity, insurance status, and urbanicity. Data were collected for persons identifying as more than one race, and MOUD treatment status of multiracial persons were reported under “other” race, thereby maintaining five minimum categories for data on race (*10*). All persons with reported ethnicity as Hispanic are grouped as Hispanic, regardless of race; persons with a reported race are grouped separately by race category. Groups are not mutually exclusive.

Urbanicity was determined using Rural-Urban Commuting Area Codes (RUCAs) associated with U.S. Postal Service zip codes (*11*,*12*). RUCA codes use urbanization measures, daily commuting, and population density to classify U.S. Census Bureau tracts into urban and rural areas (*11*,*12*). To identify the differences between core urban areas and their peripheries, the 33 RUCA codes were aggregated into the categories of urban core, other urban, and rural (Supplementary Table, https://stacks.cdc.gov/view/cdc/126230) (*11*).

These analyses were performed at the pregnancy-episode level because certain characteristics could change between pregnancies. Results might include data for more than one pregnancy per person; however, multiple-gestation pregnancies were only accounted for once in this analysis.

Differences in characteristics by MOUD treatment status were examined by chi-square tests with the Rao-Scott correction for categorical variables and regression using Taylor series linearization for continuous variables to account for clustering by persons with more than one pregnancy and by clinical site. Statistically significant differences between MOUD and non-MOUD groups were indicated if p values were <0.05. SAS (version 9.4; SAS Institute) was used for data analysis. The data cleaning and analysis processes were internally replicated by an independent analyst to correct any errors and minimize bias.

### Evaluation of Surveillance System

After the data schemas and infrastructure were built and initial pilot data were received, CDC conducted a formal evaluation using the CDC Guidelines for Evaluating Public Health Surveillance Systems (*13*,*14*). The evaluation consisted of two parts: 1) a review of existing documentation on the surveillance system and 2) key informant interviews with collaborators (*13*). Existing documentation included a published manuscript describing the rationale for the surveillance system and internal documentation including data collection tools, standard operating procedures, IT architecture diagrams, funding proposals, and pilot data (*4*). Sixteen key informant interviews were conducted, representing obstetricians, pediatricians, research scientists, and informaticians at the initial four clinical sites; data informatics contractors; CDC clinicians, epidemiologists, and health scientists working on MAT-LINK; a scientist from the CDC MAT-LINK Steering Committee; and the primary funder, ASPE’s OS-PCORTF. Interviews were conducted by a program evaluator without previous engagement in MAT-LINK who used semi-structured guides for each interview that were tailored to the relevant components of CDC Guidelines for Evaluating Public Health Surveillance Systems. A thematic analysis of interviews was performed for each evaluation component. The interview guides are available at https://stacks.cdc.gov/view/cdc/126231.

## Results

### Population Characteristics

The MAT-LINK surveillance network analyzed selected demographic characteristics of pregnant persons overall and by MOUD treatment status from seven clinical sites providing data on pregnancies during 2014–2021. Of 5,541 pregnancies, 5,626 unique pregnant person–infant dyads were identified. This analysis assessed maternal characteristics across pregnancies. Among the 5,541 reported pregnancies at the seven clinical sites, 4,381 (79.1%) included persons who received MOUD (i.e., receipt of buprenorphine with or without naloxone, methadone, or naltrexone during pregnancy) and 1,160 (20.9%) included persons who did not receive any MOUD at any point during pregnancy (Table 2). The mean maternal age across all pregnancies was 29.7 (SD = ±5.1) years. Maternal race distribution was 4,607 (86.3%) White, 312 (5.8%) Black, 250 (4.7%) other races, 141 (2.6%) AI/AN, and 26 (<1.0%) Asian or NH/OPI. Among pregnant persons, 1,372 (25.4%) were Hispanic. Insurance status across various pregnancies was 4,474 (81.6%) public insurance (e.g., Medicaid), 874 (15.9%) private insurance, 125 (2.3%) no insurance, and seven (<1.0%) other insurance (including unspecified health insurance types). On the basis of RUCA code categorization using reported zip codes, 4,615 persons (84.4%) lived in or within reasonable commuting distance to urbanized areas (i.e., ≥30% of commuting flow to an area with a population of ≥50,000). Statistically significant differences were observed for all demographic characteristics except ethnicity and urbanicity evaluated between pregnant persons receiving and not receiving MOUD. Those receiving MOUD during pregnancy were more likely to be White and older and to have public insurance.

**TABLE 2:** Maternal demographic characteristics, by medication for opioid use disorder treatment status — Maternal and Infant Network to Understand Outcomes Associated with Medication for Opioid Use Disorder During Pregnancy (MAT-LINK), seven clinical sites, 2014–2021

Characteristic	MOUD treatment* No. (%)	No MOUD treatment* No. (%)	Total* No. (%)	p value
**Maternal age, yrs, mean^†^**	30.0	28.7	**29.7**	<0.0001^§^
**Maternal race^¶^**				<0.0001**
American Indian or Alaska Native	108 (2.5)	33 (3.0)	**141 (2.6)**	
Asian or Native Hawaiian or other Pacific Islander	16 (<1.0)	10 (<1.0)	**26 (<1.0)**	
Black or African American	202 (4.7)	110 (10.2)	**312 (5.8)**	
White	3,738 (87.9)	869 (80.3)	**4,607 (86.3)**	
Other races^††^	190 (4.5)	60 (5.5)	**250 (4.7)**	
**Maternal ethnicity^¶^**				0.37**
Hispanic or Latino	1,078 (25.1)	294 (26.5)	**1,372 (25.4)**	
Not Hispanic or Latino	3,209 (74.9)	815 (73.5)	**4,024 (74.6)**	
**Maternal insurance status at delivery**				<0.0001**
Public	3,670 (84.6)	804 (70.5)	**4,474 (81.6)**	
Private	579 (13.3)	295 (25.9)	**874 (15.9)**	
No insurance	86 (2.0)	39 (3.4)	**125 (2.3)**	
Other^§§^	4 (<1.0)	3 (<1.0)	**7 (<1.0)**	
**Urbanicity^¶¶^**				0.56**
Urban core	3,651 (84.3)	964 (85.2)	**4,615 (84.4)**	
Urban other	373 (8.6)	98 (8.7)	**471 (8.6)**	
Rural	309 (7.1)	70 (6.2)	**379 (6.9)**	

**Abbreviation:** MOUD = medication for opioid use disorder.

* MOUD treatment is buprenorphine with or without naloxone, methadone, or naltrexone at any time during pregnancy. MOUD treatment: n = 4,381 (79.1%); no MOUD treatment: n = 1,160 (20.9%); total: N = 5,541 (100%). To provide a comparison group representative of pregnant persons with opioid use disorder who did not receive MOUD, clinical sites were asked to include extracted data on all pregnant persons with an active diagnosis of opioid use disorder during pregnancy who did not receive MOUD.

^†^ SD for mean maternal age: MOUD treatment = 5.0; no MOUD treatment = 5.4; total = 5.1.

^§^ Linear regression via the Taylor series linearization approach comparing mean age by MOUD treatment.

^¶^ All persons with reported ethnicity as Hispanic or Latino are grouped as Hispanic, regardless of race, and persons with a reported race are grouped separately by race category. Groups are not mutually exclusive.

** Rao-Scott chi-square test comparing maternal characteristics by MOUD treatment.

^††^ Other race includes multiracial persons and unspecified other race.

^§§^ Other insurance includes unspecified health insurance types.

^¶¶^ Urbanicity status was determined based on Rural-Urban Commuting Area designation. Subgroup descriptions are detailed in Supplementary Table at https://stacks.cdc.gov/view/cdc/126230.

### Evaluation of Surveillance System

#### Simplicity

The simplicity of a public health surveillance system refers to both its structure and ease of operation. Surveillance systems should be as simple as possible while still meeting their objectives (*13*). As an exposure-based surveillance system, MAT-LINK requires data on multiple outcomes. Data sources must be aggregated at each clinical site, making data collection and management complex. However, technological solutions (e.g., automated extraction from EHRs and tailored software) helped to simplify data collection and management. Clinical sites reported difficulties in extracting data, tracking changes to schemas, and understanding the completeness of data submitted. CDC has worked to automate processes to improve the simplicity of the system and develop central tracking and completeness reporting.

#### Flexibility

A flexible public health surveillance system can adapt to changing information needs or operating conditions with little additional time, personnel, or allocated funds (*13*). Flexibility was demonstrated by the ability to add new clinical sites and variables with relative ease. Because of the data structure and collection tools, adding new clinical sites would require minimal changes to MAT-LINK. Although the staff members at clinical sites initially perceived that the extraction process would be difficult, they discovered that the extraction tools were relatively easy to use because the tools only required minimal knowledge of Microsoft Windows Command Prompt and had detailed standard operating procedures. Clinical knowledge was required for abstraction, and staff members at the clinical sites already had such expertise. The follow-up period for pregnant persons or children could be extended easily without the need for additional data architecture. Variables such as other treatment modalities could be added to MAT-LINK as needed, although development of new data collection instruments might be required.

#### Data Quality

Data quality reflects the completeness and validity of the data recorded in the public health surveillance system (*13*). EHRs are a primary source for diagnosis and treatment information. Challenges to the quality of the data collected for MAT-LINK included completeness and correctness of data within EHRs as well as the accuracy of extraction queries and abstraction. These challenges could not be directly assessed with the limited pilot data available during the evaluation; however, quality assurance measures were assessed. The data processing steps described previously provided multiple opportunities for quality assurance. All interviewed collaborators agreed that MAT-LINK data were high quality. Staff members interviewed at clinical sites suggested that data quality might be improved by standardizing the values for missing variables, aligning the data dictionary with the data extractor validator, and providing additional trainings to standardize the interpretation of variables. Since the evaluation, the MAT-LINK team has instituted the suggested improvements.

#### Acceptability

Acceptability reflects the willingness of persons and organizations to participate in the surveillance system (*13*). Principal investigators, project managers, and data team leads from the four initial clinical sites who were interviewed expressed interest in the MAT-LINK project, although expectations about the amount of time and resources needed to collect the large number of MAT-LINK variables were not met. Certain respondents said they believed they underestimated the time needed for extraction of data by three times, which resulted in underbudgeting. Project staff members at clinical sites wanted to reduce the difficulty of data collection by aligning all variables collected with specific research questions and eliminating variables that were not necessary. In response to these findings, approximately 10% of MAT-LINK variables were changed from required to optional.

#### Sensitivity

For MAT-LINK, sensitivity relates to the ability to detect pregnancies that meet inclusion criteria (*13*). Staff members at clinical sites and subject matter experts expressed that MAT-LINK was comprehensive and captured all relevant procedures, diagnoses, conditions, treatments, and encounters. Pregnant persons without OUD but who might have opioid dependence, such as those being treated for chronic pain with opioids, might not be captured by MAT-LINK because a diagnosis of OUD was required for inclusion. During their data reviews, principal investigators and data managers made note of pregnancies with data that did not seem consistent with OUD and reviewed these charts to determine whether they met the inclusion criteria; pregnancies that were included on the basis of opioid use and not opioid use disorder were removed from the data set. In addition, data about certain pregnant persons with OUD who did not receive prenatal care from the clinical sites but delivered in their hospitals might not have been captured. A concern of a retrospective surveillance system is survival bias. Initial case ascertainment data from the initial four clinical sites indicated that a larger-than-expected proportion of pregnancies reported ended in live births, which suggests a lack of parity in the ascertainment of non–live-birth pregnancy outcomes.

#### Predictive Value Positive

Predictive value positive (PVP) is the proportion of reported cases that actually have the health-related event under surveillance (*13*). Pilot data were insufficient to examine the PVP of variables related to OUD diagnoses, MOUD, and birth outcomes. However, using infectious disease laboratory tests as a surrogate standard, analyses indicated a high predictive value for an ICD-9/10-CM diagnosis code for the infection. For example, the PVP for a hepatitis C virus diagnosis code was 95%.

#### Representativeness

**FIGURE 3:**
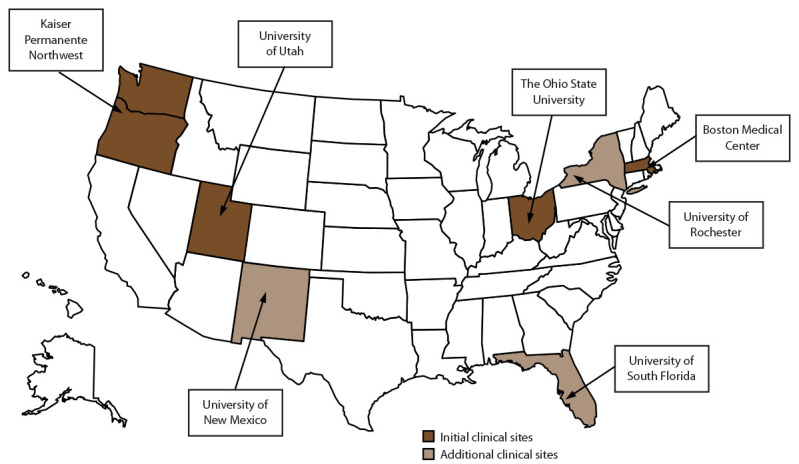
Clinical sites — Maternal and Infant Network to Understand Outcomes Associated with Medication for Opioid Use Disorder During Pregnancy (MAT-LINK), 2019–2023

Representativeness accurately describes the occurrence of a health-related event over time and its distribution in the population by place and person (*13*). MAT-LINK initially had four clinical sites, which were not representative of the entire United States; however, these clinical sites were highly representative of the pregnancies affected by OUD in their catchment areas as assessed by data provided in clinical site applications to MAT-LINK and published neonatal abstinence syndrome data. The initial four clinical sites did not represent the South or Southwest regions of the United States and had lower representation from Black (7.5%), Hispanic, (6.6%), and uninsured (<1.0%) populations compared with the overall U.S. population (*15*). The expansion to three more clinical sites added representation from the South and Southwest and increased the proportions of persons who identified as Hispanic and those without insurance (Table 2) (Figure 3).

#### Timeliness

 Timeliness reflects the speed between steps in a public health surveillance system (*13*). MAT-LINK included deliveries as early as 2014; therefore, long-term outcomes at 6 years can be evaluated immediately for dyads with a pregnancy outcome before early 2017 because the children were aged 6 years in early 2023. In addition, automated extraction and processing facilitates timely analysis and dissemination of results compared with efforts that rely fully on abstraction and manual processing.

#### Stability

Stability refers to the reliability (i.e., the ability to collect, manage, and provide data properly without failure) and availability (i.e., the ability to be operational when it is needed) of the public health surveillance system (*13*). MAT-LINK used systems managed by CDC’s Infrastructure Services Branch. This provision allowed systems to be highly stable and receive prioritized support.

#### Informatics System and Service Quality

MAT-LINK used modern data architecture to securely collect, process, manage, analyze, and share data. The database architecture used various artifacts (e.g., tables, views, and stored procedures) to store, validate, process, and retrieve data. Clinical sites submitted data inquiries securely through SharePoint. Critical issues that prevented a clinical site from moving forward with surveillance work typically were resolved the same day. Noncritical issues were sometimes resolved the same day, although solutions were scheduled or bundled with other changes. Staff members at participating clinical sites expressed appreciation for the support they received from PHII and CDC partners. Monthly meetings for data managers and individual clinical site calls were additional channels for resolving project issues and supported collaboration among the clinical sites.

#### Informatics Interoperability

Standard coded data were collected including SNOMED, ICD, CPT, RxNorm, and CVX codes, and all data were captured electronically. The CDC team developed a plan for returning clean data to clinical sites and making the data available to other researchers in compliance with the Assurance of Confidentiality.

## Discussion

### MAT-LINK Surveillance Methods

MAT-LINK is the first surveillance system to collect comprehensive, linked dyad-based data related to MOUD during pregnancy from multiple clinical sites across the United States. Data are ascertained directly from EHRs and multiple data sources within the clinical setting. MAT-LINK can obtain comprehensive data while maintaining strict confidentiality through robust IT architecture. Multiple layers of data validation are key to data quality and control. The data flow is automated from clinical sites through integration with CDC’s Secure Access Management Services system and SQL servers and REDCap. In the future, data will be available through multiple outputs, including data visualizations, public use data sets, publications, and recommendations. MAT-LINK’s architecture was designed to be interoperable and modifiable for use in other projects.

Additional strengths of the MAT-LINK project include collaboration among the clinical sites, CDC, and PHII. Clinical sites were engaged in decision-making related to the variables, piloting the IT infrastructure, and communicating feedback. User support documents were created, including a data dictionary, data extractor, REDCap user manual, standard operating procedure guides (e.g., instructions for data submissions, generating and validating XML files, and responding to data inquiries), and user acceptance test scripts to support timely onboarding and implementation of new clinical sites.

The MAT-LINK development approach was complex. Multiple components were being built in parallel that, ideally, would have occurred in phases. Key staff members were involved intermittently with CDC’s COVID-19 pandemic response, and processes were delayed. Aggressive project timelines necessitated by funding required development and staff feedback to occur simultaneously, resulting in software development delays. While the analytical plan was developed, variables changed, which complicated the data submission pilot phase.

### Selected Population Characteristics

Statistically significant differences were observed in most demographic characteristics between persons receiving and not receiving MOUD. Persons receiving MOUD during pregnancy were more likely to be White and older. Persons not receiving MOUD tended to be younger and have private insurance. No significant ethnic and urban-rural classification differences were observed between persons receiving and not receiving MOUD during pregnancy.

The demographic findings are consistent with those in other populations (*16*). The sample size in this surveillance system will provide important information to support care for persons with OUD during pregnancy.

### Surveillance Evaluation

Data from the initial four clinical sites were less representative than the overall study data, and the addition of three clinical sites improved representativeness with respect to geography, race and ethnicity, and insurance status. However, even with seven clinical sites from across the United States, the representativeness of the data remains limited. As clinical sites become increasingly skilled in data abstraction and extraction, the timeliness of the system is likely to improve.

## Limitations

The findings in this report are subject to at least five limitations. First, these are preliminary data and data collection and cleaning are ongoing; therefore, future analyses might have slight differences in selected population characteristics.

Second, outcome data were not available for analysis in this report because chart review and abstraction are ongoing due to the limitations of EHR capacities. However, these preliminary findings present an initial description of the MAT-LINK population that can be expanded upon in future analyses.

Third, whereas controlled medical vocabularies were used where possible, including RxNorm, LOINC, and SNOMED CT, not all data fit easily into the available categories. Standardization was difficult across clinical sites because of a lack of discrete fields in EHRs or inconsistencies in how these fields were collected. These challenges meant that clinical sites could not always adhere to the same pilot data submission schedule. MAT-LINK had to allow for flexibility of data submission timelines by prioritizing data sets and allowing each clinical site to provide feedback on a submission timeline that best fit their staffing needs, which led to a delay in finalizing data collection instruments.

Fourth, certain variables, such as race and ethnicity and insurance status, are subject to misclassification or incompleteness because they are not collected in the same way across all clinical sites or even at one clinical site, and inaccuracies might occur in how they are captured in medical records. Race and ethnicity could be collected via self-report or external assessment. In addition, analyses of race and ethnicity data reflect systemic racism and implicit bias and do not indicate physiologic differences in characteristics related to substance use disorder or medication metabolism. Consideration was given to reporting combined race and ethnicity data; however, these data were presented separately to facilitate a more in-depth understanding of the study population. Insurance status reflects insurance at the time of delivery or at the start of pregnancy for membership-based health systems. Variations in insurance status throughout pregnancy and postpartum are common. Therefore, data might not be consistently reported across clinical sites, which might limit the ability to accurately make comparisons across the entire data set. Inconsistent data collection is more likely if persons delivered or received other care outside of a clinical site health system, which makes data difficult to collect and might lead to missing data for these pregnancies.

Finally, other important demographic variables (e.g., highest education level) were not included in this analysis because they are poorly documented in EHRs. This limits the understanding of the surveillance population’s demographics although future analyses might be more complete with the addition of abstracted data.

## Future Directions

The next step for the MAT-LINK system is to include further data analysis and collaboration with partners to promote lessons learned about care for persons with substance use during pregnancy. Whereas MAT-LINK surveillance of pregnancies is complicated by OUD specifically, data include other substances and findings extend beyond opioids alone. One partnership is the maternal health collaborative created by ASPE that comprises representatives from federal agencies who received OS-PCORTF funding. This collaborative provides practical experience from clinicians and epidemiologists to guide national standards for linked dyad-based data. In addition, CDC facilitated the creation of a learning collaborative for clinical site primary investigators to share data analyses as they are conducted, innovate clinical strategies, and build a network of providers and researchers. Best practices from the MAT-LINK clinical sites are being written for use in the work by others who provide care for this population.

This detailed description of MAT-LINK surveillance methods can provide information to guide other similar projects as well as pique interest in future data and analysis collaborations. The MAT-LINK protocol for data collection meets a uniform standard and can be used by any health care system to collect data on an ongoing basis to rapidly evaluate MOUD, other exposures, and outcomes to ensure that effective and safe clinical care options are prioritized.

Because of the flexibility of the system, future iterations of MAT-LINK could include expanding data collection for existing clinical sites beyond August 2021 pregnancy outcomes, following children beyond age 6 years, and adding additional clinical sites. A new funding opportunity for longitudinal surveillance of pregnant persons and their infants will build on MAT-LINK’s data infrastructure by adding more clinical sites and collecting additional data on pregnant person–infant dyads with MOUD exposure through 2027. Sustaining and expanding MAT-LINK data collection might support additional research and continued monitoring of pregnancies complicated by OUD.

## Conclusion

MAT-LINK is the first surveillance system to collect comprehensive, longitudinal, linked dyad-based data on perinatal and early childhood outcomes associated with MOUD during pregnancy from multiple clinical sites. The preliminary findings are that pregnant persons with OUD who are taking MOUD are more likely to be older and White and to have public insurance. These findings are important markers of possible differences in health care access and clinical care. The surveillance evaluation indicated that MAT-LINK has strengths that other projects can leverage and challenges from which other projects can learn. This timely and flexible system has obtained data on approximately 5,600 dyads; ongoing analyses of those and future data will provide information to support clinical and public health guidance to improve health outcomes among pregnant persons with OUD and their children.

## Footnotes

* See e.g., 45 C.F.R. part 46.102(l)(2), 21 C.F.R. part 56; 42 U.S.C. §241(d); 5 U.S.C. §552a; 44 U.S.C. §3501 et seq.
